# Hajdu-Cheney Syndrome: A Novel NOTCH2 Mutation in a Spanish Child in Treatment with Vibrotherapy: A Case Report

**DOI:** 10.3390/jcm11175205

**Published:** 2022-09-02

**Authors:** Jonathan Cortés-Martín, Lourdes Díaz-Rodríguez, Beatriz Piqueras-Sola, Juan Carlos Sánchez-García, Antonio Liñán González, Raquel Rodríguez-Blanque

**Affiliations:** 1 Research Group CTS1068, Andalusia Research Plan, Junta de Andalucía, 18014 Granada, Spain; 2 School of Nursing, Faculty of Health Sciences, University of Granada, 18071 Granada, Spain; 3 Hospital University Virgen de las Nieves, 18014 Granada, Spain; 4 School of Nursing, Faculty of Health Sciences, Melilla Campus, University of Granada, 52005 Melilla, Spain

**Keywords:** Hajdu-Cheney syndrome, rare diseases, acroosteolysis, osteoporosis, bone resorption, NOTCH2

## Abstract

A case report of an 11-year-old boy with a de novo variant in NOTCH2 and clinical features characteristic of Hajdu-Cheney syndrome is reported, with acroosteolysis of the distal phalanges of the feet and hands, generalized osteoporosis, musculoskeletal and craniofacial alterations, short stature, bowing of long bones, vertebral anomalies, genu recurvatum, hypertrichosis, joint and skin hyperlaxity, atopic dermatitis, megalocorneas, micrognathia and frequent respiratory infections, among others. Treatment is with bisphosphonates in the framework of bone density improvement and with focal vibration therapy for rehabilitation of the musculoskeletal system and gait improvement. The three generalities of this pathology—phenotypic variability, degenerative character and the presence of generalized osteoporosis and acroosteolysis of the distal phalanges—are seen in this case, whose diagnostic confirmation was made by genetic study.

## 1. Introduction

Hajdu-Cheney Syndrome (HCS) is classified as a rare disease, which like 80% of this type of pathologies is of genetic origin [1]. It responds to other names, such as Acro-dento-osteo-dysplasia; acroosteolysis with osteoporosis and changes in the skull and jaw; arthodentroosteodysplasia; and serpentine fibula syndrome and polycystic kidneys. It is referenced as ORPHA955 in ORPHANET [2] and #102500 in the OMIM database [3]. This syndrome mainly affects connective tissue and belongs to the group of acroosteolytic syndromes [4].

The prevalence of this disease is less than one person in one million (<1/1,000,000) [5] and is caused by a heterozygous mutation in the NOTCH2 gene [6], specifically on chromosome 1p13–p11. Although sporadic cases are observed [7], the pattern of inheritance is autosomally dominant [8].

Approximately 100 cases have been described in the scientific literature since the first description of the disease by N. Hajdu in 1948 [9]. Later, in 1965, D. Cheney completed the description with his study [10]. In all the cases described, the same general characteristics are observed: the degenerative character [11], phenotypic variability [12] and a picture of osteolysis of the distal phalanges and generalized osteoporosis [13], accompanied by other clinical manifestations.

The worsening of the disease over time increases the levels of dependence and disability in these types of patients [1].

The variability of expression of the mutated gene gives rise to different phenotypes for the same syndrome, so it is difficult to find all the clinical manifestations in the same person [11]. 

The osteolysis of the distal phalanges [14] and generalized osteoporosis seen in all patients diagnosed with this disease [13] are accompanied by a series of clinical manifestations.

The clinical spectrum is very broad, ranging from cranial alterations [9] such as the presence of Wormian bones, prominent occipital crest, dolichocephaly, batrocephaly and absence of frontal sinuses, among others. Coarse facial features such as hypertelorism, synofridism, low-set ears, arched palate, loss of teeth [15] and deep voice, among others, stand out. At the skeletal level, kyphosis and scoliosis, joint hyperlaxity and short stature are seen [16]. This syndrome affects the respiratory system with frequent infections, as well as the digestive system and the renal system [17]. Congenital heart disease [18] is another common manifestation of the pathology. Motor development, speech and hearing are also compromised. Inguinal and abdominal hernias, as well as plantar ulcers, are peculiarities of the syndrome. The association of polycystic kidneys and serpentine fibula is characteristic of this disease [19]. The most frequent complications are basilar invagination [20,21] and ventilatory restriction due to rib cage malformation.

The diagnostic orientation is made by observation of the phenotype and radiological controls [22]; it is true that there are clinical links with other diseases with which a differential diagnosis should be approached. These diseases with which a differential diagnosis should sometimes be made are Scleroderma, Progeria and Alaguille syndrome, among others [23]. In any case, the definitive diagnosis should be by genetic study [24].

Current treatment for this disease is aimed at addressing the clinical complications and correcting the problems that appear as the pathology evolves, highlighting all types of care aimed at maintaining and improving the quality of life of these patients [25]. At present, despite scientific advances on the subject, there is no effective or curative treatment for this rare disease [26].

## 2. Patient Information

The patient is an 11-year-old male of Caucasian origin. He is a student and properly vaccinated. He was born from a controlled pregnancy and normal delivery. He is the son of healthy parents and the youngest of three siblings. There is no family history of the same pathology. His brother of 21 years old has been operated on for cholesteatomas. His brother of 27 years old was diagnosed with spastic paraparesis due to a sporadic mutation of SPG4. 

**Figure 1:**
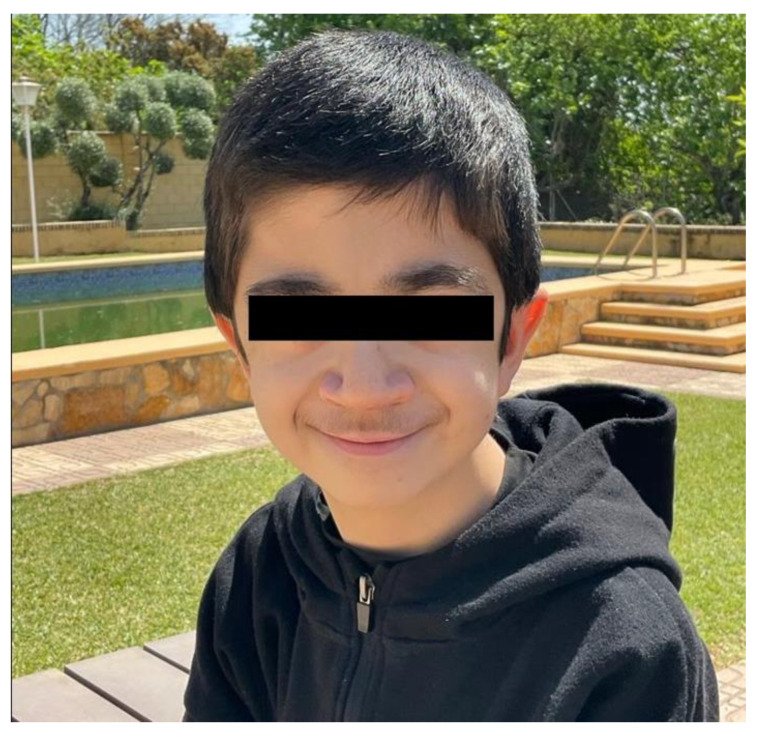
Current photograph of the patient.

Genetically diagnosed with Hajdu-Cheney syndrome, he presents acroosteolysis of distal phalanges of feet and hands, generalized osteoporosis, cranial malformations, presence of Wormian bones, coarse facial features, short stature, joint hyperlaxity, recurrent respiratory infections and musculoskeletal alterations. The following picture (Figure 1) is a current photo of the patient.

The child was born in April 2011; pregnancy and delivery were normal, despite the diagnosis of gestational diabetes that was treated with insulin, without incident. Newborn weight was 4340 g, height was 54 cm and head circumference was 36 cm.

The mother was 44 years old, with a height of 171.3 cm, with menarche at the age of 15 years. She had two spontaneous abortions during her fertile life. The father was 45 years old and 173.8 cm tall. Both are without relevant pathologies. Maternal grandparents are second cousins. The family genogram can be found in Figure S1 of the Supplementary Material.

No anomalies were observed at birth. In the development of the first months of life, some hypotonia in the lower extremities and a resting posture of the neck in flexion were observed. Based on aspects such as these, in addition to the family history related to genetic diseases, it was decided to request a medical opinion on the patient’s health status.

The first clinical record of the case already showed a delay in the closure of the posterior fontanel, which still persists, and a delay in dentition and language. He started walking at 9 months of age. Short distal phalanges, ribs with keeled deformity and Wormian bones in lambdoid suture were identified. At the cardiac and auditory level, no incidences were detected in the first few months. There was good growth, weight and height.

Based on the clinical manifestations presented by the patient, an in-depth study was carried out by different medical specialties to identify a diagnosis that justified the phenotype and the clinical manifestations.

The following physical examinations and evaluations reflect the rapid evolution of the syndrome, and the findings found in conjunction with the clinical presentation meet a typical description of Hajdu-Cheney Syndrome.

Osteolysis was seen in the distal phalanges of the hands and feet with deformity, highlighting the shortening of the fourth and fifth metacarpals. Cranial palpation with slight occipital prominence at the level of the parietal suture, retromicrognatia and prominent sternum were seen. Other aspects to highlight in the first explorations were the diastasis of the supraumbilical rectum, tubiform thorax and pectus carinatum; genu recurvatum and valgus flatfoot; joint hyperlaxity and generalized cutaneous hyperlaxity, more noticeable in the elbow; mild hypotonia, but limiting in the day-to-day; delay and alterations in the order of dentition; atopic dermatitis, slight hypertrichosis, hard and dry hair, as well as abnormally populated eyebrows. Features such as ogival palate, full and slightly drooping cheeks and wide philtrum stand out. Ocular proptosis was observed with blue and megalocorneal sclerae. Colds and respiratory infections, such as pneumonia, were frequent. Cardiopulmonary study and neurological evaluation were normal, as was psychomotor development. At 2 years and 6 months, the weight was 12 kg, height 89.7 cm and head circumference 50 cm. The diagnosis was based on clinical observation, the study of the phenotype presented and the radiological findings. In May 2013, a genetic study was performed to confirm the origin of the pathology.

The genetic test performed was the sequencing of exon 34 of the NOTCH2 gene, by means of a blood extraction.

The procedure consists of the extraction of genomic DNA from the sample; PCR (polymerase chain reaction) amplification of the coding region, as well as the flanking intronic regions of exon 34 of the NOTCH2 gene was performed. DNA sequencing and capillary electrophoresis reactions were prepared. Bioinformatic analysis of the sequences was obtained by comparison with the reference sequence NG_008163.1. 

A molecular study was performed for the analysis of small deletions/insertions and point mutations in the coding region and splicing sites of exon 34 of the NOTCH2 gene.

By direct analysis of the NOTCH2 gene, a heterozygous 18-nucleotide deletion (c.6446_6463del) was detected in the sample. This deletion presumably causes a change in the reading pattern and the appearance of a premature stop codon (p.Ser2149) at the protein level. 

**Figure 2:**
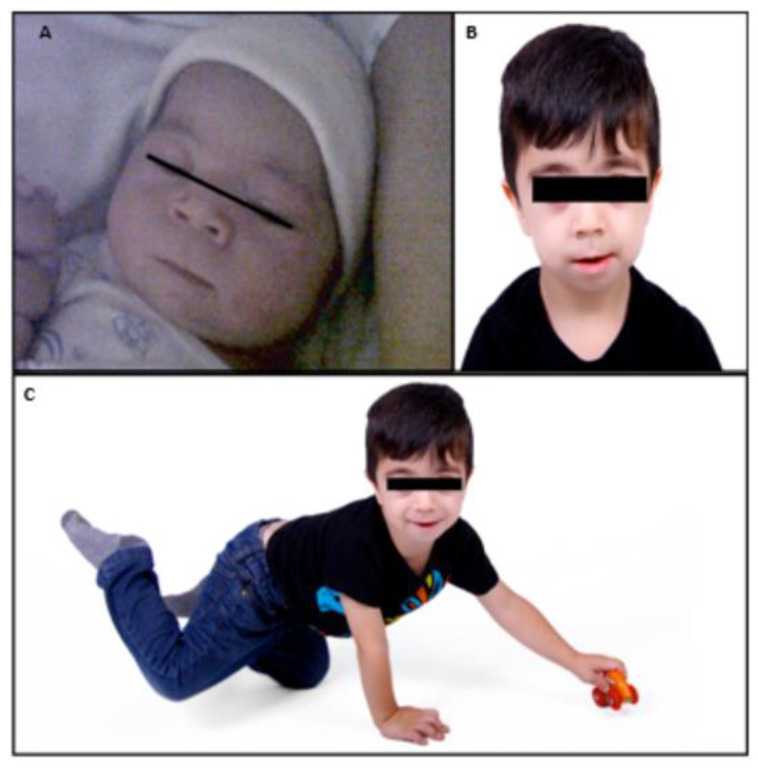
Childhood photos. (**A**) Newborn. (**B**) Facial features, 4 years old. (**C**) Full body, 4 years.

Once diagnosed, the clinical follow-up was performed in a multidisciplinary manner between the different medical specialties that best adapt to the needs and problems that arise during the development of the disease. The description of the case will be made by analyzing in detail the different clinical manifestations framed within its functional space. The following image, (Figure 2) are photos of the patient’s childhood.

## 3. Musculoskeletal Features

There are several musculoskeletal disorders. In the first evaluations, complete radiological controls were carried out and repeated over time for identification and follow-up. In these diagnostic tests, different characteristic findings of the disease were observed, such as wide sutures with multiple Wormian bones and elongated sella turcica, at the cranial level; osteolysis of the distal phalanges of hands and feet, retraction of the distal metaphysis of the radius, widened distal metaphysis of tibia and fibula and of coarse aspects; hip dysmetries, scoliosis, flattening of D6 and biconcave vertebrae.

Peculiar facial features were also observed, with a mildly keeled thorax with tendency for lumbar kyphosis and scoliosis with the lower hip, with the axial axis slightly lateralized to the right. Thick hands with short distal phalanges and flexible flat feet of 4°, the left one bigger. Telemetry showed a right scoliotic attitude with a 5 mm hip descent. 

Gait control was performed, and although it was correct and allowed him to jump and run, the use of corrective insoles was proposed. 

The last radiological findings identified were a fracture of the fifth metatarsal in 2016 and a vertebral fracture in 2021.

In 2017, cervical protrusion was detected, which becomes more evident when coughing; after that, cervical ultrasound detected pulmonary herniation.

Magnetic resonance imaging was performed without finding signs of basilar invagination for the moment. A decrease in the height of the vertebral bodies of T7, T4 and T2 with vertebral biconcave morphology in relation to the sinking of the vertebral plates was observed. 

**Figure 3:**
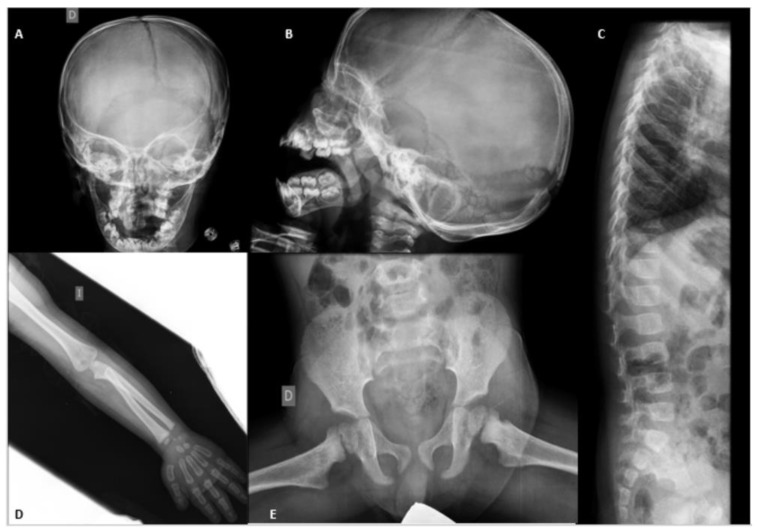

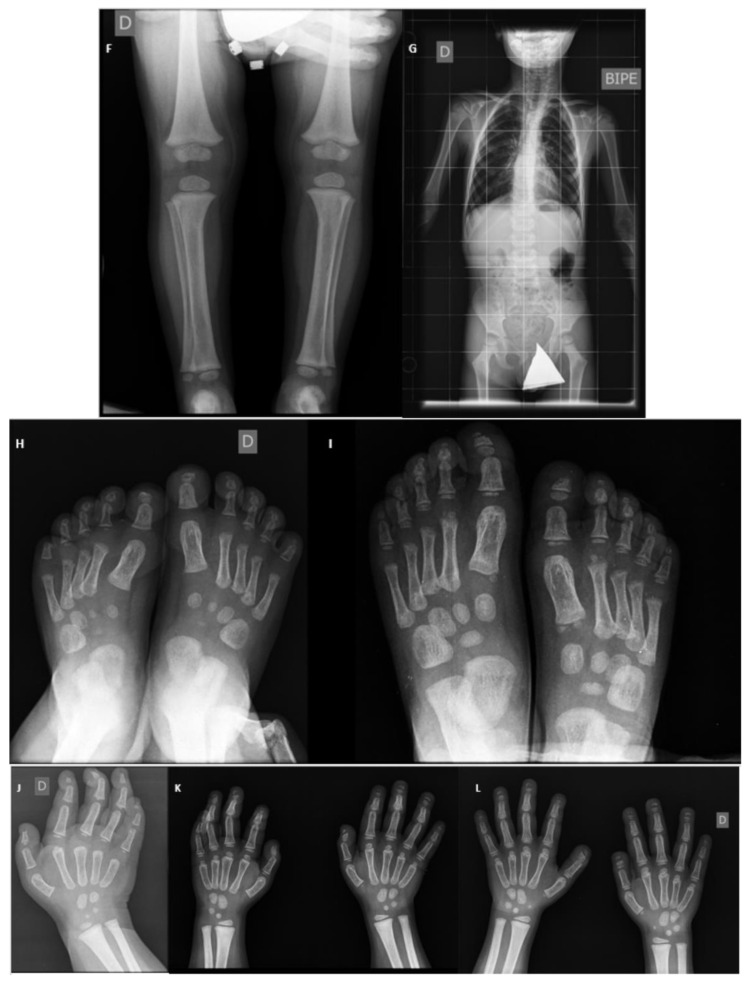
Radiological controls. (**A**) Skull, year 2012. (**B**) Lateral skull, year 2012. (**C**) Spinal column, year 2012. (**D**) Left arm, year 2013. (**E**) Pelvis, year 2013. (**F**) Legs, year 2014. (**G**) Standing position, year 2015. (**H**) Feet, year 2012. (**I**) Feet, year 2016. (**J**) Hands, year 2012. (**K**) Hands, year 2014. (**L**) Hands, year 2016.

Abdominal ultrasound with multifrequency convex probe was performed after denying drug and material allergies. The results confirmed a liver of normal size and echogenicity and a normal gallbladder and biliary tract. The pancreas and spleen were without alterations. Kidneys were well-located, with cortico-medullary differentiation without ectasia. The bladder was at half repletion without alterations in its wall. The following image, (Figure 3) are radiological controls.

## 4. Endocrine Aspects

The patient is referred to this service for growth control and osteoporosis prevention.

With regard to growth development, there is a certain delay that affects both weight and height, with most of the measurements taken being below the appropriate percentile. The following table (Table 1) shows the growth development.

For the prevention of osteoporosis, densitometries were performed continuously in order to know the progression of osteoporosis and to administer the appropriate treatment.

With the first densitometries, no treatment was required since all the parameters were adequate. In 2016, it was decided to start treatment with bisphosphonates due to the progression of the disease. Intravenous cycles of pamidronate and vitamin D were administered orally until 2020. The results improved, and it was decided to give a rest period and re-evaluate the administration of these drugs.

## 5. Respiratory Features

The patient presents respiratory noises during wakefulness and more intense ones during sleep, but without presence of nocturnal apneas. Oral breathing is predominant. He sleeps in a lateral ulna position, and in early infancy in a supine position, with neck hyperextension. Striking hyporexia was detected without previous physical effort. A cutaneous and systemic anaphylactic reaction to peanut and walnut was described, as well as an allergy to the fungus *Alternaria alternate.* He has an intolerance to lentils, manifested by abdominal pain. No known drug allergies were reported. Rhinosinus infections are frequent, with abundant mucus, cough and pneumonia. Sleep oximetry was evaluated, and the results confirmed that there is no obstructive sleep apnea syndrome. In infectious processes, gastroesophageal reflux is enhanced, without morning halitosis. He tolerates exercise well. 

## 6. Cardiological Aspects

A cardiological study was performed as a preventive measure since the description of the disease is associated with cardiac alterations. Echocardiography showed a nonhypertrophic LV (left ventricle), nondilated with normal function. There was no MR (mitral insufficiency); no gradient in LVOT (left ventricular outflow tract tachycardia); a normal RV (right ventricle); and no IT (tricuspid insufficiency). A systolic murmur was detected. The sinus rhythm’s frequency is in normal ranges. The patient is normotensive.

In 2018, a jugular protrusion was observed at the jugular level that increased when coughing. A CT angiography was performed. A PDA (permanent ductus arteriosus) with a length of 12 mm was detected. The aortic end measured 3.6 × 2.7 mm; and the pulmonary end, with a filiform passage, measured 0.6 mm. The right jugular vein was dilated and tortuous. The PDA had no hemodynamic repercussions. In 2021, percutaneous closure was performed and the evolution was favorable.

## 7. Neurosurgery Aspects

He was referred to this service for evaluation for craniofacial malformation syndrome due to Wormian bones and Chiari propensity. After one year of age, hand deformity and delayed lambdoid closure with increased spaces in the lambdoid sutures appeared. Radiological control was performed. A metopic ridge, lower-third hypoplasia, low-set ears and fusion defects in lambdoid sutures were seen, as well as hypertrichosis in the dorsal and lumbar regions. There were no fusion defects in the posterior arches. The fingers had short and wide phalanges, which were also in the feet, and were more striking in the first finger. Craniospinal MRI and renal ultrasound were requested to evaluate associated malformations.

## 8. Neurology Aspects

At one and a half years of age, a study was carried out in this service, which showed an autonomous gait from 12 months of age and adequate crawling from 9 months of age. Handling was good and he used a mother–dad language. The neonatal screening was normal, with no incidences. Hearing was normal, although the test was repeated twice due to doubts. Gait was somewhat unsteady, but muscle strength and mass were normal. The myotatic reflexes had a slight increase in the reflexogenic area in the thighs. CPR (plantar cutaneous response) was observed in flexors. There were no signs of cerebellar dysfunction. There was hypotonia, probably related to mild joint hyperlaxity.

At 2 years and 11 months, the disease continued to progress normally. There was some delay in expressive language, with abundant jargon but the ability to make a sentence and good verbal comprehension. He would receive support in his school and treatment with a speech therapist. He was referred to the early care service to perform a Battelle test.

## 9. Early Care

**Figure 4:**
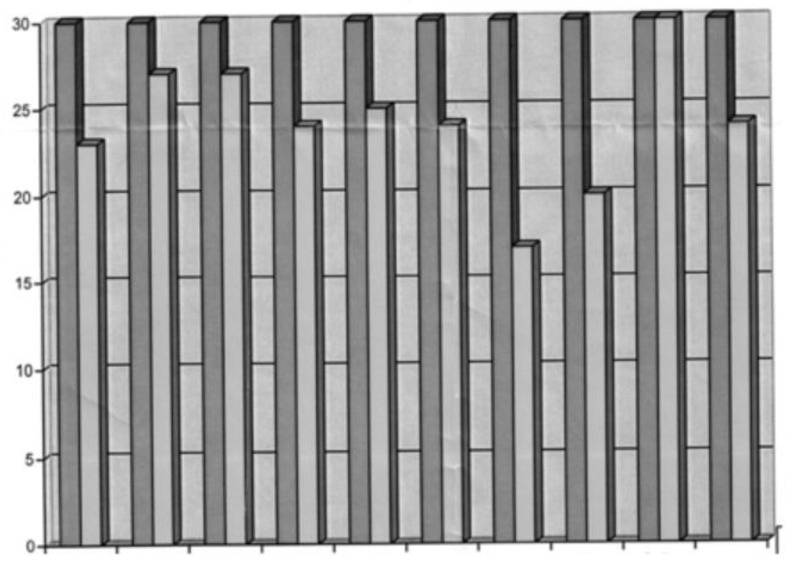
Battelle test graph.

A screening test, Battelle [27], was performed at 32 months. It is a tool aimed at assessing global development in several specific areas. There were no deficits in the adaptive, gross motor, fine motor and cognitive areas. He was at −1 standard deviation from the measurement in the total motor area and at −1 standard deviation from the measurement in the personal social, receptive and total areas. He was more than −2 standard deviations away from the measurement in the areas of expressive and total communication. All are shown below in Figure 4 and Table 2.

Currently, the evolution of language is favorable; he improves in communication and is able to make sentences. He presents some dyslalia. He has good social interaction.

## 10. Ophthalmological Aspects

In this service, a study is performed to identify possible pathological findings at ocular level. A fundus examination was performed, where ocular proptosis with blue and megalocorneal sclerae was confirmed. In addition, a vision control was performed, detecting 5 diopters in the left eye and 5.5 in the right eye. He needs glasses for correction.

## 11. Otorrine Aspects

**Figure 5:**
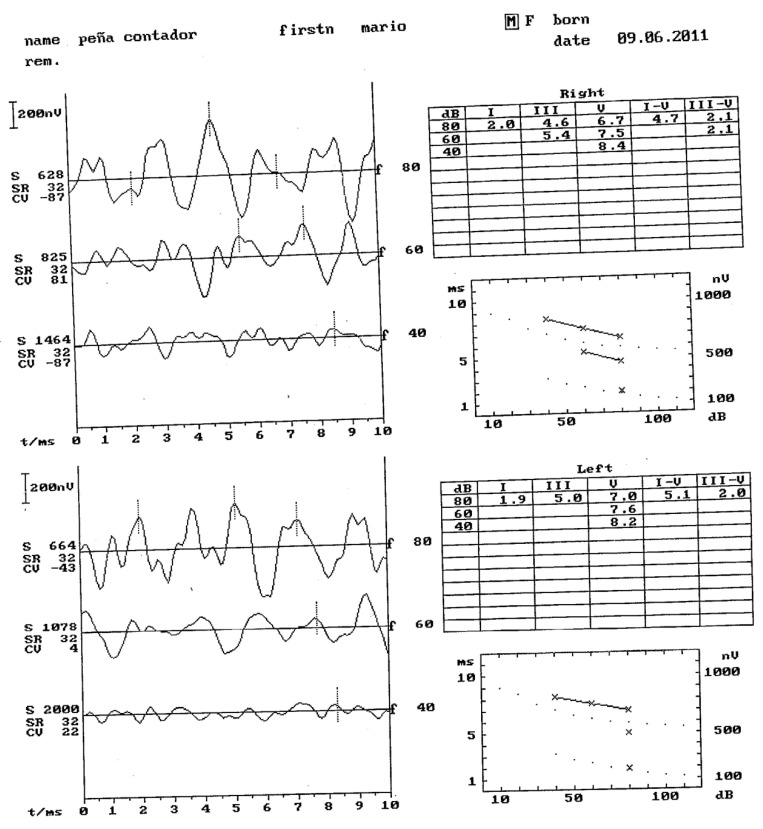
Auditory potentials.

The patient underwent a study in this service because he presented repeated upper respiratory catarrh. He was a nocturnal snorer, without apneas but with characteristic postures. An otoscopy was performed, where wax plugs were observed in both ears. In the study of the oral cavity and oropharynx, there were grade IV tonsils (narrow isthmus of the fauces). He underwent surgery on two occasions for placement of auditory drains. In the second intervention, they were left permanently. His vegetations were also operated on by this service. The auditory potentials do not show signs of hearing loss at the moment. The results are shown in Figure 5.

## 12. Maxillofacial Features

The teeth began to erupt at 4 years of age, with some alteration in their alignment. The total eruption of the teeth was not complete until 7 years of age. At the age of 10, the removal of teeth was necessary to facilitate the eruption of the permanent dentition.

## 13. Gait Study

A gait study was performed. On visual examination, a bilateral Trendelenburg gait or “waddling gait” was observed. In addition, a significant valgus of the feet was also visually observed, which is very evident when barefoot. There is no difficulty in muscle recruitment, except when rising from the horizontal position. There, he shows difficulty in abdominal recruitment and other groups of the trunk flexion pattern, compensating in the activity with the help of the arms to perform it successfully. No sensory alterations have been observed in fine or gross sensibility, as well as in proprioception. Reflexes are present and symmetrical, with low response. There are no dysmetria or coordination problems.

There is a very pronounced valgus in both feet, which when weight is shifted monopodally does not allow an ideal alignment, so the reaction forces of the ground allow a straightening reaction in this type of support. 

In view of the above, it was decided to treat the muscular hypotonia by means of focal vibration axially on the trunk muscles, hippotherapy and work in the swimming pool.

After the focal vibration treatment, a significant improvement in gait was observed, with a gait without Trendelenburg and with a faster step cadence and longer steps, as can be seen in Video S1.

## 14. Psychosocial Aspects and Lifestyle

At present, the patient is perfectly adapted to his environment. He has a structured and functional family, and is integrated in the educational center. He attends the academic course corresponding to his age. He enjoys his group of friends and has developed social skills. He has correct handling and use of technology. He attends musical percussion classes at levels more advanced than his age and has a highly developed sense of rhythm and music.

## 15. Milestones

Table 3 shows the main milestones of the case described above.

## 16. Prognosis

The prognosis of patients affected by HCS will depend on the severity of the disease, clinical complications and the degenerative evolution of each patient. Generalized osteoporosis and the development of acroosteolysis will lead to fractures, difficulty in ambulation and dependence for daily living activities. The most frequent complications in this disease are basilar invagination, which will lead to neurological alterations or thoracic deformity causing ventilatory restriction.

## 17. Therapeutic Intervention

The therapeutic intervention in this case can be divided into several lines of action.

Regarding the pharmacological line, treatment with bisphosphonates for the improvement and prevention of osteoporosis is worth mentioning. In addition, vitamin D was administered for the same purpose. All of the underlying problems of this disease that appeared were treated with existing conventional pharmacological treatments: analgesia for pain; antibiotic therapy for infectious processes; bronchodilators for respiratory processes.

Surgical treatment in this case was performed to treat PDA, and the placement of ear drains and vegetations was identified during its clinical course.

The self-care recommendations are aimed at maintaining physical and intellectual activity as much as possible and avoiding overweight as a preventive method against the development of the disease.

A pioneering treatment for this type of patient is focal vibration (vibrotherapy). This consists of the application of a type of mechanical vibration by means of a specific vibration apparatus at a superficial level on tendons or muscles. Electronic vibrators can be used to modify different treatment parameters such as frequency, amplitude or pressure. It serves as an agent of stimulation of muscular and cutaneous mechanoreceptors within the framework of movement reprogramming. It represents an excellent passive re-education tool due to its action on the active elements of the joint [28]. 

In this case, the patient has limitations in certain activities such as changing and maintaining the position of the body. He is able to perform the usual daily transfers without assistance, but needs support on joining his upper limbs to sit from a supine position. In addition, he is able to move without difficulty in all types of environments, although the gait pattern is accentuated in situations of effort or uneven terrain. He has the ability to run at low speed.

As seen in Video S1, the patient’s gait improves after the vibrotherapy session. As seen in the image, on the left side the patient is presented before the session and on the right side after the session.

Therefore, gait improvement is essential for this patient. Focal vibration is the treatment of choice for this purpose. It is also used in patients with muscular spasticity diagnosed with cerebral palsy.

## 18. Discussion

One of the main problems observed in the world of rare diseases is the delay in diagnosis [29]. This has a negative impact on the clinical approach to the disease, plunging the patient into a situation of uncertainty and chaos. On average, it can take a patient 4–5 years to obtain a diagnosis. Approximately 20% of patients take approximately 10 years to obtain a diagnosis [30]. In most cases, there is a delay in diagnosis due to the general lack of knowledge in the field of rare diseases, difficulties in accessing the necessary information and an insufficient number of professionals and specialized health centers, in addition to the low prevalence and clinical links between these pathologies.

The case described in this report does not comply with the aforementioned statistics. In this case, only 2 years were necessary to obtain a definitive diagnosis, since the phenotype and the clinical presentation were very evident.

Guidance to diagnosis is made by observation of the phenotype and clinical presentation [22]. Normally, in the process of the diagnostic phase, clinical links are found with other diseases, with which a differential diagnosis must be made [23] before starting the genetic study. This is the case of the patient described by Herrmann et al. [31], who during the diagnostic stage was suspected of pycnodysostosis [32] because of the clinical similarities that existed. 

With a concise diagnostic orientation, a genetic study was performed [24]. In the case of our patient, sequencing of exon 34 of the NOTCH2 gene was performed in 2013, by means of a blood extraction. The result was a heterozygous deletion of 18 nucleotides, which justifies the diagnosis of this syndrome [6]. Unlike other mutations described in some studies [33,34], this type of mutation had not been previously described in the spectrum of Hajdu-Cheney syndrome, so there are no previously described cases that share this variant.

The pathological picture presented by the patient described in this article brings together the three essential characteristics of Hajdu-Cheney syndrome, the phenotypic variability, the degenerative character and the picture of generalized osteoporosis and acroosteolysis of the distal phalanges.

Due to the variability of NOTCH2 expression, there is a variability in the phenotypes of this type of patient that becomes evident when making comparisons with other described cases. The patients described by Swan et al. [35], Ades et al. [36] and Takatani et al. [37] stand out, where differences in physical and clinical appearance are appreciated, despite having the same diagnosis.

The degenerative character is identified in most of the cases published in the scientific literature. One of the most obvious examples is described by Harnasch H. [38]. It is true that for the observation of the degenerative character there must be a follow-up over time, an aspect that with the age of our patient is complicated to verify. In spite of the 11 years of the case presented, degenerative nuances observed at a musculoskeletal level are identified in the radiological findings in the same way that they are seen in the cases described in pediatric patients [34]. Table S1 of the Supplementary Material contains a list of the most relevant published cases of this disease diagnosed in pediatric age (less than 15 years old).

The acroosteolysis of the distal phalanges and generalized osteoporosis described are present in the majority of diagnosed cases of this syndrome, highlighting the cases of Rosenmann et al. [39], Elias et al. [40] and Bruckner et al. [41], where they justify that the most prevalent signs are acroosteolysis and generalized osteoporosis.

At the musculoskeletal level, in addition to osteoporosis and acroosteolysis, craniofacial alterations are observed, as in the cases described by Letchumanan et al. [14], Stathopoulos et al. [42] and Nunziata et al. [43]. Biconcave vertebrae are also seen in the cases described by Vissarionov et al. [44] and Chawla [45].

Deformities in hands are observed, as in the cases described by Jiménez et al. [46], Shurtleff et al. [47], Brown et al. [13] and Ventosa et al. [48] in feet, highlighting this finding in the patients of Greenberg et al. [49] and Colmenares Roldán et al. [50].

The genu recurvatum presented by the patient was previously described on different occasions by authors such as Williams [51] and Weleber et al. [52].

Kyphosis and scoliosis hinder gait, an aspect that is described in the patient of Rosenmann et al. [39], forcing our patient to use corrective insoles and vibrotherapy treatment to improve gait. Another aspect that our case also shares with the case of Rosenmann et al. [39] is the presentation of the nails in watch glass.

Growth retardation was justified in the report of Siklar et al. [16], who described the relationship between growth hormone and short stature in these patients.

Regarding the frequent rhinosinus infections presented by our patient, cough and pneumonia processes can be observed in the cases of Williams [51] and Sasaki [53]. 

Congenital heart disease is frequently found in the clinic of this syndrome, as in the case of Sargin et al. [18]. In one of the examinations, our patient was found to have a systolic murmur, and later, by means of an Angiotac, he was diagnosed with PDA.

The dentition abnormalities present in this case are also seen in the cases of Shaw [54] and Lee et al. [55]. Also highlighted are the studies of Bazopoulou-Kyrkanidou et al. [15] and Antoniades et al. [56] on dental alterations. Another significant work on dental restorations in patients with this syndrome was that of Vingerhoedt et al. [57].

It should be noted that aspects such as food allergies to peanuts, walnuts or lentils and the *Alternaria alternate* fungus have not been previously described in any of the cases published on this disease, in addition to the optical characteristic presented by the patient, the blue sclerae.

There is currently no curative and effective treatment for this pathology. It is true that there are studies that address this aspect, namely those by Sakka et al. [58] and Pittaway et al. [26], who worked with biosphonates, those being the treatment of choice in the case described. Specifically, the drug of choice for our patient was Pamidronate, in the same way as for the patients of Tsinopoulou et al. [59] and Al-Mayouf et al. [60].

At the surgical level, the work of Murtagh-Schaffer et al. [61] on spinal reconstructions is noteworthy, which has some relation with the signs detected in our patient at the structural level.

In this case, self-care recommendations are proposed, such as maintaining physical and intellectual activity as much as possible and avoiding overweight as a preventive method against the development of the disease. 

For postural hygiene and gait improvement, our patient has been treated with vibrotherapy with very positive results. This therapy is not applied as the treatment of choice in any of the cases described in the scientific literature. It is a possible line of future research for the development of this pathology.

The current treatment for HCS is aimed at addressing the complications and underlying problems, offering an improvement in quality of life and life expectancy [25].

This study reflects the existence of the three generalities that characterize Hajdu-Cheney syndrome. In the last 70 years, only about 100 diagnosed cases of this rare disease have been described. In view of the low prevalence of the syndrome, the description of confirmed and updated cases such as the one presented today is essential. 

In particular, the contribution of this case to the scientific literature and to the development of the disease is based on the presentation of a mutation never described before. This new variant adds two previously undescribed aspects to the disease phenotype: bluish sclerae and the presence of allergies with systemic repercussions. However, it is true that with the description of only one case with these features, it is too early to speak of their inclusion in the definitive phenotype of the syndrome.

Another aspect that deserves attention is the choice of vibrotherapy as a treatment for gait reeducation in this type of patient with muscular hypotonia.

In spite of the existing advances, a complete and updated description of the phenotype of this disease, including a large sample, is still necessary.

## Figures and Tables

**Table 1:** Growth development.

DATE	WEIGHT kg	SIZE cm
Newborn	4.340	54
2 years and 6 months	12	89.7
5 years	15.5	105
6 years and 8 months	18	117
10 years and 1 month	24.6	131.9
10 years and 8 months	26.4	134
11 years and 1 month	26.3	135.4

**Table 2:** Age equivalence by area.

	Personal-Social	Adaptive	Gross Motor	Fine Motor	Total Motor	Receptive	Expressive	Total Comunication	Cognitive	Total Score
Age equivalent in months	23	27	27	24	25	24	17	20	32	24

**Table 3:** Main milestones in the description of the case.

Birth	2011
Diagnosis	2013
Beginning of therapy with early care	2014
Use of glasses	2014
Use of corrective insoles	2015
Initiation of bisphosphonate therapy	2016
Detection of PDA	2018
Focal vibration treatment	2021

## Supplementary Materials

The following supporting information can be downloaded at: https://www.mdpi.com/article/10.3390/jcm11175205/s1. Video S1: Results after vibrotherapy session. Figure S1: Family genogram. Table S1: List of the most relevant published cases of this disease diagnosed in pediatric age (less than 15 years old). References [62,63,64,65,66,67] are cited in the supplementary materials.
