# Supplementary material for: Hajdu-Cheney Syndrome: A Novel NOTCH2 Mutation in a Spanish Child in Treatment with Vibrotherapy: A Case Report
Source: J Clin Med. 2022 Sep 2;11(17):5205. doi: 10.3390/jcm11175205 (PMC9457082; doi:10.3390/jcm11175205)
Supplement: Supplementary file 1 [file jcm-11-05205-s001.zip › jcm-1899231-supplementary.pdf]

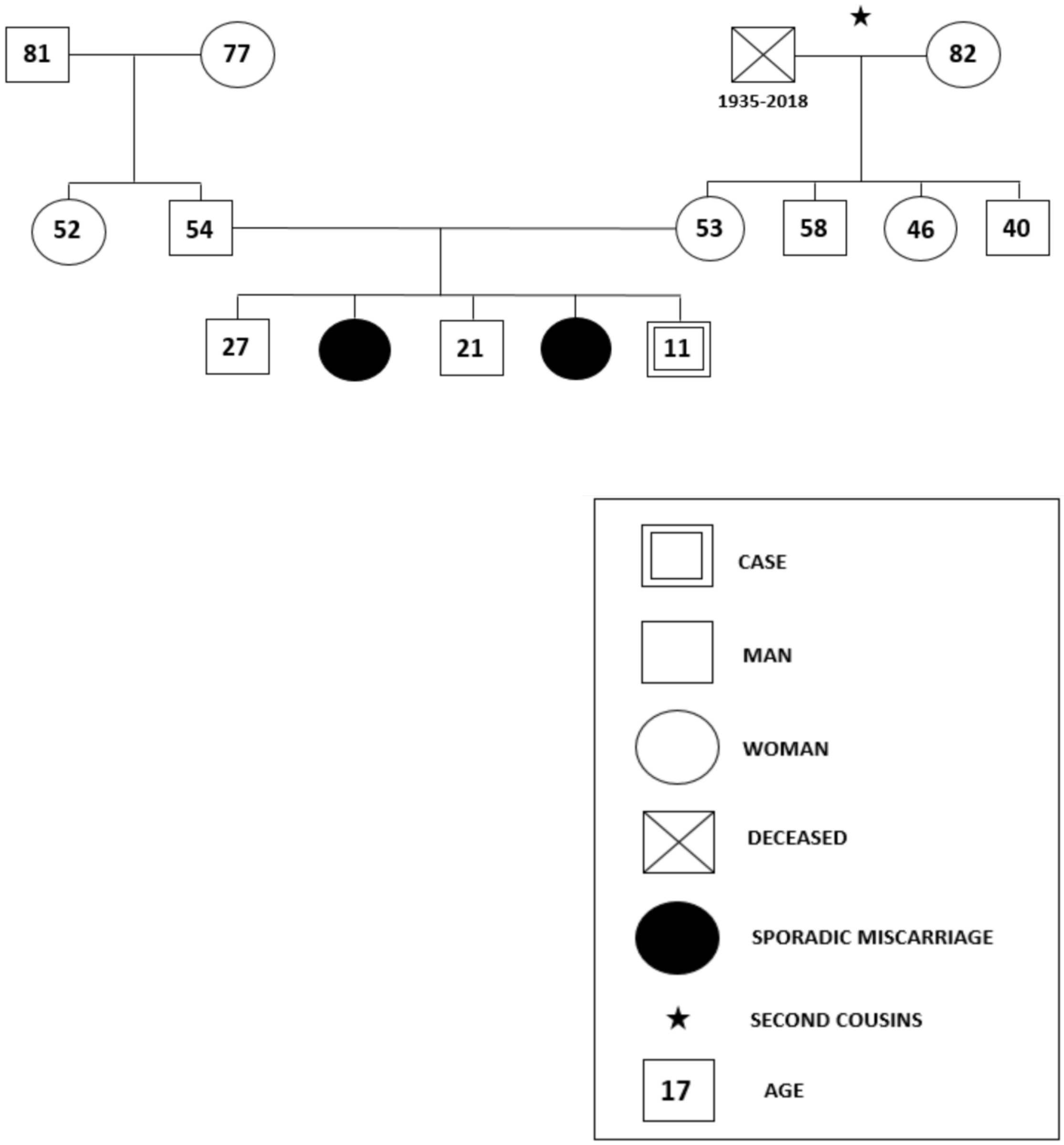

Figure S1: Family genogram.

Table S1: List of the most relevant published cases of Hajdu-Cheney syndrome in pediatric age (less than 15 years old).

AUTHOR	ARTICLE	YEAR OF PUBLICATION	PATIENT NUMBER	PATIENT´S AGE	SEX	COUNTRY
Shurtleff DB et al. [1]	*Hereditary Osteolysis With Hypertension and Nephropathy.*	1964	1	4	MALE	UNITED STATES
			2	6	FEMALE	UNITED STATES
			3	8	FEMALE	UNITED STATES
Herrmann et al. [2]	*Arthro-Dento-Osteo Dysplasia (Hajdu-Cheney Syndrome)*	1973	4	10	MALE	UNITED STATES
Brown et al. [3]	*The acro-osteolysis syndrome: Morphologic and biochemical studies.*	1976	5	7	FEMALE	UNITED STATES
Weleber et al. [4]	*The Hajdu-Cheney syndrome Report of two cases and review of the literatura.*	1976	6	12	FEMALE	UNITED STATES
			7	12	FEMALE	UNITED STATES
Rosenmann et al. [5]	*Sporadic Idiopathic Acro-Osteolysis with Cranio-Skeletal Dysplasia, Polycystic Kidneys and Glomerulonephritis A case of the Hajdu-Cheney syndrome.*	1977	8	15	MALE	ISRAEL
Elias et al. [6]	*Hereditary Osteodysplasia with Acre-Osteolysis (The Hajdu-Cheney Syndrome).*	1978	9	12	MALE	UNITED STATES
Zeman et al. [7]	*Hajdu-Cheney syndrome in a 3½ year old girl.*	1994	10	3.5	FEMALE	AUSTRALIA
Brennan et al. [8]	*Hajdu-Cheney Syndrome: Evolution of Phenotype and Clinical Problems.*	2001	11	5	FEMALE	UNITED STATES
			12	5	FEMALE	UNITED STATES
Antoniades et al. [9]	*Hajdu-Cheney syndrome (acro-osteolysis): A case report of dental interest.*	2003	13	9	MALE	GREECE

August et al. [10]	*Anesthesia for a child with Hajdu-Cheney syndrome.*	2009	14	8	FEMALE	UNITED STATES
Yamaguchi et al. [11]	*A case report of anesthesia for a child with Hajdu–Cheney syndrome.*	2013	15	10	FEMALE	JAPAN
Ventosa et al. [12]	*Acro-osteolysis in a 4 year-old patient: clinical features of Hajdu-Cheney syndrome.*	2013	16	4	MALE	SPAIN
Battelino et al. [13]	*End-Stage Renal Disease in an Infant With Hajdu-Cheney Syndrome.*	2016	17	8	MALE	SLOVENIA
Sakka et al. [14]	*Bone Structural Characteristics and Response to Bisphosphonate Treatment in Children With Hajdu-Cheney Syndrome.*	2017	18	15	MALE	UNITED KINGDOM
			19	6.8	MALE	UNITED KINGDOM
			20	7.8	FEMALE	UNITED KINGDOM
			21	10.8	FEMALE	UNITED KINGDOM
			22	10	MALE	UNITED KINGDOM
			23	13	FEMALE	UNITED KINGDOM
			24	15	MALE	UNITED KINGDOM
Ruo-Lan Gong et al. [15]	*A Novel Mutation of Notch homolog protein 2 gene in a Chinese Family with Hajdu-Cheney Syndrome.*	2017	25	4	MALE	CHINA
		2018	26	6	FEMALE	LONDON

Pittaway et al. [16]	*Bisphosphonate therapy for spinal osteoporosis in Hajdu-Cheney syndrome – new data and literature review.*		27	8	FEMALE	LONDON
			28	11	FEMALE	LONDON
			29	15	FEMALE	LONDON
Graversen et al. [17]	*Phenotypic presentations of Hajdu-Cheney syndrome according to age – 5 distinct clinical presentations.*	2019	30	10	FEMALE	DENMARK
Jiménez et al. [18]	*Hand Deformities in Hajdu-Cheney Syndrome: A Case Series of 3 Patients Across 3 Consecutive Generations.*	2020	31	6	MALE	SPAIN

Supplementary references:

1. Shurtleff, D.B.; Sparkes, R.S.; Clawson, D.K.; Guntheroth, W.G.; Mottet, N.K. Hereditary Osteolysis With Hypertension and Nephropathy. *JAMA J. Am. Med. Assoc.* **1964**, *188*, 363–368, doi:10.1001/jama.1964.03060300025005.
2. Herrmann, J.; Zugibe, F.T.; Gilbert, E.F.; Opitz, J.M. Arthro-Dento-Osteo Dysplasia (Hajdu-Cheney Syndrome). *Z. Kinderheilk* **1973**, *114*, 93–110.
3. Brown, D.M.; Bradford, D.S.; Gorlin, R.J.; Desnick, R.J.; Langer, L.O.; Jowsey, J.; Sauk, J.J. The acro-osteolysis syndrome: Morphologic and biochemical studies. *J. Pediatr.* **1976**, *88*, 573–580, doi:10.1016/S0022-3476(76)80009-1.
4. Weleber, R.G.; Rodney K. Beals The Hajdu-Cheney syndrome. Report of two cases and review of the literature. *J. Pediatr.* **1976**, *88*, 243–249.
5. Rosenmann, E.; Penchas, S.; Cohen, T.; Aviad, I. Sporadic idiopathic acro-osteolysis with cranio-skeletal dysplasia, polycystic kidneys and glomerulonephritis A case of the hajdu-cheney syndrome. *Pediatr. Radiol.* **1977**, *6*, 116–120,

doi:10.1007/BF00973535.

6. Elias, A.N.; Pinals, R.S.; Clarke Anderson, H.; Gould, L. V.; Streeten, D.H.P. Hereditary osteodysplasia with acro-osteolysis (the Hajdu-Cheney syndrome). *Am. J. Med.* **1978**, *65*, 627–636, doi:10.1016/0002-9343(78)90851-3.

7. ZEMAN, J.; HOUSTKOVA, H.; KOZLOWSKI, K. Hajdu–Cheney syndrome in a 3 1/2 year old girl. *Australas. Radiol.* **1994**, *38*, 228–230, doi:10.1111/j.1440-1673.1994.tb00183.x.

8. Brennan, A.M.; Pauli, R.M. Hajdu-Cheney syndrome: Evolution of phenotype and clinical problems. *Am. J. Med. Genet.* **2001**, *100*, 292–310, doi:10.1002/1096-8628(20010515)100:4<292::AID-AJMG1308>3.0.CO;2-4.

9. Antoniades, K.; Kaklamanos, E.; Kavadia, S.; Hatzistilianou, M.; Antoniades, V. Hajdu-Cheney syndrome (acro-osteolysis): A case report of dental interest. *Oral Surg. Oral Med. Oral Pathol. Oral Radiol. Endod.* **2003**, *95*, 725–731, doi:10.1067/moe.2003.151.

10. August, D.A.; Ramos, D.C. Anesthesia for a child with Hajdu-Cheney syndrome. *Paediatr. Anaesth.* 2009, *19*, 649–650.

11. Yamaguchi, S.; Nakamura, K.; Takahashi, Y. A case report of anesthesia for a child with Hajdu-Cheney syndrome. *J. Anesth.* **2013**, *27*, 949–950, doi:10.1007/s00540-013-1642-4.

12. Ventosa, P.; Gorina, N.; Balaguer Santamaria, A.; Riera, L.; Casals, I. Acroosteólisis en un paciente de 4 años: datos clínicos del síndrome de Hajdu-Cheney. *An. Pediatr.* **2013**, *79*, 62–63, doi:10.1016/j.anpedi.2012.11.025.

13. Battelino, N.; Writzl, K.; Bratanič, N.; Irving, M.D.; Novljan, G. End-Stage Renal Disease in an Infant With Hajdu-Cheney Syndrome. *Ther. Apher. Dial.* **2016**, *20*, 318–321, doi:10.1111/1744-9987.12444.

14. Sakka, S.; Gafni, R.I.; Davies, J.H.; Clarke, B.; Tebben, P.; Samuels, M.; Saraff, V.; Klaushofer, K.; Fratzl-Zelman, N.; Roschger, P.; et al. Bone Structural Characteristics and Response to Bisphosphonate Treatment in Children With Hajdu-Cheney Syndrome. *J. Clin. Endocrinol. Metab.* **2017**, *102*, 4163–4172, doi:10.1210/jc.2017-01102.

15. Gong, R.L.; Wu, J.; Chen, T.X. A Novel Mutation of Notch homolog protein 2 gene in a Chinese Family with Hajdu-Cheney Syndrome. *Chin. Med. J. (Engl).* **2017**, *130*, 2883–2884, doi:10.4103/0366-6999.219141.

16. Pittaway, J.F.H.; Harrison, C.; Rhee, Y.; Holder-Espinasse, M.; Fryer, A.E.; Cundy, T.; Drake, W.M.; Irving, M.D. Bisphosphonate therapy for spinal osteoporosis in Hajdu-Cheney syndrome - New data and literature review. *Orphanet J. Rare Dis.* 2018, *13*.

17. Graversen, L.; Handrup, M.M.; Irving, M.; Hove, H.; Diness, B.R.; Risom, L.; Svaneby, D.; Aagaard, M.M.; Vogel, I.; Gjørup, H.; et al. Phenotypic presentations of Hajdu-Cheney syndrome according to age – 5 distinct clinical presentations. *Eur. J. Med. Genet.* **2019**, *63*, doi:10.1016/j.ejmg.2019.04.007.

18. Jiménez, I.; Medina-Gontier, J.; Caballero, J.; Medina, J. Hand Deformities in Hajdu-Cheney Syndrome: A Case Series of 3 Patients Across 3 Consecutive Generations. *J. Hand Surg. Am.* **2020**, doi:10.1016/j.jhsa.2020.02.012.
